# Biallelic variants in *COPB1* cause a novel, severe intellectual disability syndrome with cataracts and variable microcephaly

**DOI:** 10.1186/s13073-021-00850-w

**Published:** 2021-02-25

**Authors:** William L. Macken, Annie Godwin, Gabrielle Wheway, Karen Stals, Liliya Nazlamova, Sian Ellard, Ahmed Alfares, Taghrid Aloraini, Lamia AlSubaie, Majid Alfadhel, Sulaiman Alajaji, Htoo A. Wai, Jay Self, Andrew G. L. Douglas, Alexander P. Kao, Matthew Guille, Diana Baralle

**Affiliations:** 1 Wessex Clinical Genetics Service, Princess Anne Hospital, University Hospital Southampton NHS Foundation Trust, Coxford Rd, Southampton, SO165YA UK; 2 European Xenopus Resource Centre, University of Portsmouth School of Biological Sciences, King Henry Building, King Henry I Street, Portsmouth, PO1 2DY UK; 3 Faculty of Medicine, University of Southampton, Duthie Building, Southampton General Hospital, Tremona Road, Southampton, SO16 6YD UK; 4 Exeter Genomics Laboratory, Level 3 RILD building, Royal Devon & Exeter NHS Foundation Trust, Barrack Road, Exeter, EX2 5DW UK; 5 University of Exeter Medical School, RILD building, Royal Devon & Exeter NHS Foundation Trust, Barrack Road, Exeter, EX2 5DW UK; 6 Department of Pediatrics, College of Medicine, Qassim University, Qassim, Saudi Arabia; 7 Department of Pathology and Laboratory Medicine, King Abdulaziz Medical City, Riyadh, Saudi Arabia; 8 Division of Genetics, Department of Pediatrics, King Abdullah Specialized Children Hospital, King Abdulaziz Medical City, Ministry of National Guard Health Affairs (MNGHA), Riyadh, Saudi Arabia; 9 King Abdullah International Medical Research Centre, Ministry of National Guard Health Affairs (MNGHA), Riyadh, Saudi Arabia; 10 King Saud bin Abdulaziz University for Health Sciences, Ministry of National Guard Health Affairs (MNGHA), Riyadh, Saudi Arabia; 11 Division of Allergy and Clinical Immunology, Department of Pediatrics, King Abdullah Specialized Children Hospital, King Abdulaziz Medical City, Ministry of National Guard Health Affairs (MNGHA), Riyadh, Saudi Arabia; 12 Zeiss Global Centre, School of Mechanical and Design Engineering, University of Portsmouth, Portsmouth, PO1 3DJ UK

**Keywords:** COPB1, COPI, β-COP, Microcephaly, Cataract, *Xenopus* model, Coatomer, Intellectual disability

## Abstract

**Background:**

Coat protein complex 1 (COPI) is integral in the sorting and retrograde trafficking of proteins and lipids from the Golgi apparatus to the endoplasmic reticulum (ER). In recent years, coat proteins have been implicated in human diseases known collectively as “coatopathies”.

**Methods:**

Whole exome or genome sequencing of two families with a neuro-developmental syndrome, variable microcephaly and cataracts revealed biallelic variants in *COPB1*, which encodes the beta-subunit of COPI (β-COP). To investigate Family 1’s splice donor site variant, we undertook patient blood RNA studies and CRISPR/Cas9 modelling of this variant in a homologous region of the *Xenopus tropicalis* genome. To investigate Family 2’s missense variant, we studied cellular phenotypes of human retinal epithelium and embryonic kidney cell lines transfected with a *COPB1* expression vector into which we had introduced Family 2’s mutation.

**Results:**

We present a new recessive coatopathy typified by severe developmental delay and cataracts and variable microcephaly. A homozygous splice donor site variant in Family 1 results in two aberrant transcripts, one of which causes skipping of exon 8 in *COPB1* pre-mRNA, and a 36 amino acid in-frame deletion, resulting in the loss of a motif at a small interaction interface between β-COP and β’-COP. *Xenopus tropicalis* animals with a homologous mutation, introduced by CRISPR/Cas9 genome editing, recapitulate features of the human syndrome including microcephaly and cataracts. In vitro modelling of the *COPB1* c.1651T>G p.Phe551Val variant in Family 2 identifies defective Golgi to ER recycling of this mutant β-COP, with the mutant protein being retarded in the Golgi.

**Conclusions:**

This adds to the growing body of evidence that COPI subunits are essential in brain development and human health and underlines the utility of exome and genome sequencing coupled with *Xenopus tropicalis* CRISPR/Cas modelling for the identification and characterisation of novel rare disease genes.

**Supplementary Information:**

The online version contains supplementary material available at 10.1186/s13073-021-00850-w.

## Background

The endomembrane system is composed of a group of organelles (including the endoplasmic reticulum (ER), Golgi apparatus and endoplasmic reticulum-Golgi intermediate compartment (ERGIC)). It allows modification, packaging and transport of proteins through the secretory pathway in eukaryotic cells [1]. Proteins move between different organelles in membrane-bound transport vesicles coated with proteins. Protein coat complexes include coatomer protein I (COPI) complex, coatomer protein II (COPII) complex, a number of adaptor protein (AP) complexes including clathrin adaptor protein complexes, and the retromer complex [2]. These protein coats select cargo (proteins and lipids) for transport and facilitate transport vesicle formation. The functioning of protein coats is a fundamental cellular process. Knockout of certain subunits of COPI and COPII, such as *COPB2*, in mice is embryonic lethal [3, 4]. Depletion of COPI in cancer cells results in decreased cell survival, impaired autophagy and ER stress [5].

**Fig. 1:**
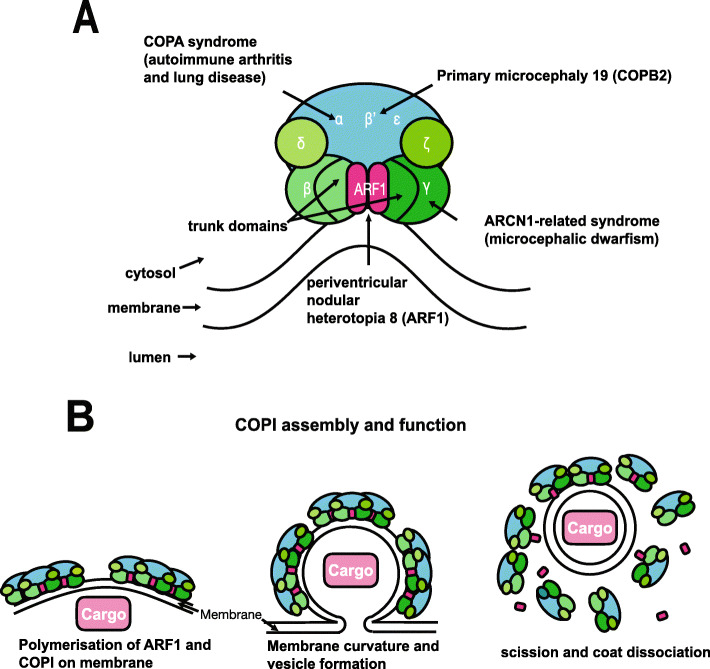
Structure and function of the COP1 complex. **a** COPI consists of a scaffold “B-subcomplex” (blue) and an adaptor “F-subcomplex” (green). When GTP-bound, two ARF1 small GTPase molecules associate with the membrane and bind COPI via the β-COP and γ-COP subunits. A number of subunits of this complex have been implicated in human disease as shown. **b** COPI complexes and their ARF1 molecules associate into triads. Cargo, such as ER-resident proteins which need to be returned from the Golgi, are selected by binding directly with COPI subunits or indirectly through transmembrane receptors which in turn bind with COPI. COPI polymerises on the membrane enabling its deformation/curvature, and eventually budding and scission of the transport vesicle. When released, the vesicle’s coat is shed and ARF1 and COPI dissociate. Adapted from Nickel et al. [12]

COPI consists of seven subunits (α, β (the focus of this study), β’, γ, δ, ε and ζ) (Fig. 1a) [6]. The COPI subunits can be divided into the F- (or Adaptor-like) subcomplex (containing the β, γ, δ and ζ subunits) and the B- (or Cage-like) subcomplex (α, β′ and ε) (Fig. 1a) [7–9]. The adaptor-like subcomplex, which contains β-COP, is analogous to the clathrin adaptor AP subcomplexes [10]. Both β-COP and γ-COP contain an α-solenoid “trunk” domain, connected by a linker to a small appendage domain [11].

ARF1, a small GTPase, catalyses COPI recruitment to the vesicle. When GTP-bound, ARF1 becomes stabilised in the membrane and initiates recruitment of COPI through binding with the trunk domains of the β-COP and γ-COP subunits [13]. COPI causes increased curvature of the membrane, until a vesicle forms and scission occurs [14]. After the vesicle is released, the COPI coat is shed and ARF1 and COPI can dissociate (Fig. 1b).

The primary functions of COPI are returning ER-resident proteins back from the Golgi to the ER, recycling certain transmembrane cargo receptors from the *cis-*golgi and ERGIC, and intra-Golgi retrograde transport [15]. COPI is also involved in lipid droplet formation and lipolysis [16]. Depletion of β-COP results in defective cargo transport and defective compartmentalisation of the ERGIC trans-Golgi network, Golgi, and recycling endosomes, emphasising its importance in both compartmentalisation and transport [17].

As with other coat protein complexes, several elements of the COPI machinery have been linked to human disease. The term “coatopathies” has been suggested to describe this heterogenous group of conditions (Fig. 1a) [2]. Homozygous variants in *COPB2* have been linked to a severe microcephaly syndrome (MIM # 617800) [4]. The COPI complex may also play a role in Alzheimer disease as it is involved in the localisation of amyloid precursor protein and silencing of δ-COP leads to decreased amyloid plaques and improved memory in mice [18]. Interestingly, loss-of-function mutations in *ARCN1* (which encodes δ-COP) cause a distinctive syndrome of craniofacial abnormalities, microcephalic dwarfism and mild developmental delay (MIM # 617164) [19]. Loss-of-function variants in *COPA* cause an autoimmune disorder of arthritis and lung disease (MIM # 616414) [20, 21]. Hypomorphic variants in *ARF1* (which though not part of COPI, interacts closely with it, as described above) have been linked to brain malformations including periventricular heterotopia (MIM # 618185) [22]. β-COP has never been associated with Mendelian disease.

This study aimed to identify the molecular cause of a syndromic presentation in two unrelated families and investigate the pathogenicity of putative variants. We describe a new genetic disorder identified in six individuals across two families associated with biallelic variants in *COPB1*. This syndrome is typified by a severe intellectual disability with variable microcephaly and cataracts.

## Methods

### Subjects and sequencing

As part of clinical care, Exeter Clinical Laboratory performed whole exome sequencing on lymphocyte-extracted DNA from a duo of two affected sisters from Family 1. This involved exome sequence analysis of the coding region and conserved splice sites of 23,244 genes by next-generation sequencing (Twist Core Human Exome/Illumina NextSeq). The duo analysis utilised a gene-agnostic approach to identify rare homozygous or compound heterozygous variants shared by both sisters. Segregation analysis of a candidate variant was undertaken on lymphocyte-extracted DNA from all family members in keeping with routine clinical practice. As this investigation identified a splicing variant in a non-morbid gene, the participants were recruited to a research study to investigate the functional effect of these variants using lymphocyte-extracted RNA. Patient enrolment took place at University Hospital Southampton UK.

Family 2 were recruited as part of a multicentre clinical genome study in consanguineous Saudi populations [23]. Whole genome sequencing was performed on lymphocyte-extracted DNA and data was analysed with both gene-agnostic and gene panel approaches. The enrolment, data collection and analysis were conducted at King Abdulaziz Medical City, Riyadh, Saudi Arabia. Data collection was performed by analysing results from electronic health records. WGS was carried in clinical CAP/CLIA-accredited laboratories as part of a collaborative project. WGS was performed using HiSeq 4000. This family was identified with use of the GeneMatcher platform [24].

### Analysis of splice site variant

#### In silico analysis

The prediction tools, Human Splicing Finder, Splice Site Prediction by Neural Network, MaxEntScan and SpliceAI, were used to predict the impact of Family 1’s variant on splicing [25–28].

#### Reverse transcriptase PCR (RT-PCR)

The effect of Family 1’s variant on splicing was investigated using RT-PCR. Blood from the probands, parents and unaffected siblings was collected in PAXgene® Blood RNA Tubes (PreAnalytiX, Hombrechtikon, Switzerland). RNA was then extracted from blood samples using the PAXgene blood RNA kit (PreAnalytiX, Hombrechtikon, Switzerland) and quality control was performed using a 2100 Bioanalyzer Instrument (Agilent, Santra Clara, USA). RNA extracted from blood samples was converted to cDNA using the High-Capacity cDNA Reverse Transcription Kit (Thermo Fisher Scientific, Waltham, USA). The primer pairs were designed using Primer 3 and ordered from Integrated DNA Technologies (IDT, Coralville, USA) (COPB1_ c957+1G>T_F_CTGGTGAATGAGAAGGATGCA, COPB1_c957+1G>T_R RV=GGCTTCACGAACAAACTCCA).

PCR experiments were performed using GoTaq G2 Polymerase PCR system (Promega, Madison, USA) and separated by agarose gel electrophoresis. The amplicons were then documented under Chemidoc XRS+ (Bio-Rad, Hercules, USA). The amplicons for further analysis were cloned into plasmids using a TA cloning kit, with pCR 2.1 vector (Thermo Fisher Scientific, Waltham, USA). The plasmids carrying inserts were sent to Source Bioscience (Nottingham, UK) for Sanger sequencing.

#### *Xenopus* care

Nigerian strain *Xenopus tropicalis* were housed and maintained within the European *Xenopus* Resource Centre, University of Portsmouth, in re-circulating systems (25 °C) with 15% daily water changes on a 13–11-h light-dark cycle. All procedures were conducted in accordance with the Home Office Code of Practice, under PPL 70/8983 with approval from the University of Portsmouth’s Animal Welfare and Ethical Review Body.

Adult female *X. tropicalis* were primed the evening prior to egg collection with 10 IU hCG (Chorulon, Intervet) and received a boosting dose of 100 IU hCG the following morning. Egg clutches were obtained by gentle abdominal massage and fertilised with cryopreserved sperm. Frozen *X. tropicalis* spermatozoa were generated within the European *Xenopus* Resource Centre from 12- to 15-month-old male *X. tropicalis* exhibiting enhanced nuptial pads. Sperm cryopreservation followed a protocol adapted from Sargent and Mohun [29].

#### Generating knockout animals using CRISPR-Cas9

The exon structure of the *copb1* gene in *X. tropicalis* and humans is identical despite the difference shown currently on Xenbase’s gene page; we identified an untranslated exon contributing to the 5′ UTR of the gene by using its conservation with *X. laevis*. The target region within *copb1* exon 8 was identified using Xenbase (originally designed against v9.1 of the genome and checked against v10) [30], and two single-stranded oligonucleotides were selected based on the following criteria: high mutagenic activity, minimal predicted off-target events (CRISPRscan) [31] and a high frameshift frequency (indelphi) [32].

sgRNA3-taatacgactcactataGGACTCGCTCATGGGAAGGGgttttagagctagaa

sgRNA4-taatacgactcactataGGAGCACTCGCTCATGGGAAgttttagagctagaa

Single guide RNAs (sgRNA) were synthesised from single-stranded oligonucleotides (Invitrogen, above) containing the T7 promoter as described in the Taq-based method by Nakayama et al., using the T7 megashortscript kit (Invitrogen) [33]. sgRNAs were subsequently purified (SigmaSpin post-reaction clean up columns) and visualised by agarose gel electrophoresis. The quantity and purity were assessed using a NanoDrop 1000 spectrophotometer and sgRNAs were stored at − 80 °C as single use aliquots. Each sgRNA (500 pg) was co-injected with Cas9 protein (2.6 ng, spy Cas9 NLS, NEB) at 1-cell and 2-cell stages into *X. tropicalis* embryos from both a wild-type strain and a transgenic line ([Xtr.Tg (tubb2b:GFP)Amaya] RRID:EXRC_3001) [34]. Although we show un-injected control tadpoles, these experiments were performed alongside CRISPR-based knockouts of other genes and the phenotypes shown here were specific to the *copb1* sgRNA(s).

To determine the efficiency of indel formation, the target region of interest was amplified from genomic DNA (Lysis Buffer: 50 mM Tris (pH 8.5), 1 mM EDTA, 0.5% [v/v] Tween-20, 100 μg/ml Proteinase K) by polymerase chain reaction, using primers designed in Primer3 (http://primer3.ut.ee; Exon8 FWD: TGTGTCCCTAGGCAGCCG, Exon8 REV: CGAGGACACCAGTTCCAGT).

A T7 Endonuclease I (NEB) assay was used to indicate success of indel formation and this was confirmed using Sanger sequencing (Genewiz) [34, 35]. Due to the results seen as a consequence of the Family 1 mutation, we tested whether exon skipping had occurred in these samples. cDNA was prepared using Tristar solution [36] from control and crispant tadpoles; PCR products (amplified using primers spanning Exon6-7:CTACAAGGTTTGCCATGCCAAC and Exon9-10:GATGACCAGCTCTTCCACGTTG) were subcloned into pGEMTeasy and compared by Sanger sequencing (Genewiz).

#### Phenotypic analysis of knockout tadpoles

Gross morphological differences between un-injected tadpoles and crispant tadpoles were identified using a Zeiss Axio Zoom.V16 Stereomicroscope (with fluorescence for GFP-expressing animals). Detailed structural differences were examined by high-resolution microcomputed tomography (MicroCT). For bright-field/fluorescence microscopy, tadpoles were anaesthetised in 0.025% MS222, whilst tadpoles for MicroCT were fixed in paraformaldehyde following the method described for wholemount in situ hybridisation of *Xenopus* embryos [37]. These fixed specimens were immediately contrast stained in phosphotungstic acid (PTA) following an adapted protocol from Metscher [38]. Briefly, samples were stained in 1% PTA (in water) for 24 h, washed in methanol and placed in 1% PTA for a further 24 h. Specimens were allowed to rest in methanol for 1 week, before samples were embedded in 0.7% agarose within a 20 μl pipette tip (to prevent movement during imaging). Samples were imaged using the ZEISS Xradia Versa 520 (Carl Zeiss Microscopy, Pleasanton, CA, USA) set to operate at a voltage of 50 kV and a current of 75 mA. A × 4 objective lens was used, resulting in an effective isotropic voxel size of 3.1 μm. In total, 1601 projections were collected over 360° with an exposure time of 2.0 s per projection. Each tomogram was reconstructed to 16-bit grey-level images using the manufacturer’s software (Scout and Scan Reconstructor, Carl Zeiss Microscopy, Pleasanton, CA, USA) which employs a filtered back projection algorithm. The imaged volumes were then visualised using TXM3DViewer (Carl Zeiss Microscopy, Pleasanton, CA, USA).

### Analysis of missense variants

#### In silico analysis

SIFT, PolyPhen 2 and CADD tools were used to predict the impact of Family 2’s variant on β-COP protein structure and function [39–42]. Conservation at the nucleotide level was assessed using the PhyloP score which attempts to measure deviation from the neutral rate of substitution including conservation and acceleration [43]. ConSurf conservation scores were used to measure the evolutionary rate of the amino acid [44]. PyMOL (Schrodinger) package was used to model the effect of the missense variant on the 3D structure of β-COP in complex with other components of COPI coat complex with the ArfGAP2 uncoating factor (pdb file 5NZS).

#### Site-directed mutagenesis of *COPB1* constructs

Specific patient mutation was introduced into a Myc-DDK-tagged *COPB1* transcript 1 (NM_016451) expression vector (Origene, MD, USA) using the Q5 Site-Directed Mutagenesis Kit (New England BioLabs, MA, USA) following manufacturer’s instructions with customised primers designed using NEB Base Changer [45] (COPB1_ c.1651_T>G _F GGATGGAGATgTCTTTGTTGC, COPB1_ c.1651_T>G _R AGAAGGAATCCTCTCAAG).

Plasmids were purified using the QIAprep Spin Miniprep Kit (Qiagen, Hilden, Germany) and successful mutagenesis was confirmed with Sanger sequencing by Source Bioscience (Nottingham, UK) using custom sequencing primers (COPB1_c.1651_T>G TGAGGCACAGGGAAATTCGA).

Transfection-grade plasmid preparation was then performed with the QIAprep Spin Midiprep Kit (Qiagen, Hilden, Germany).

#### Culture of human cell lines

hTERT RPE-1 cells (immortalised human retinal pigmentary epithelial cells (American Type Culture Collection, Manassas, VA, USA)) were maintained in a 50/50 mix of Dulbecco’s Modified Eagles Medium (DMEM) and Ham’s F12 (DMEM/F12) at 37 °C 5% CO_2_. Human embryonic kidney 293 cells (HEK 293 cells (American Type Culture Collection, Manassas, VA, USA)) were maintained in high glucose DMEM at 37 °C 5% CO_2_.

#### Nucleofection/transfection of cell lines

hTERT-RPE1 cells were nucleofected with mutant plasmid or wild type (WT) plasmid using the Nucleofector 4D (Lonza) and P3 kit (Lonza), following manufacturer’s instructions. HEK293 cells were transfected with mutant or WT plasmid using PEI.

#### Immunofluorescence staining and confocal imaging

Forty-eight hours after nucleofection, hTERT-RPE1 cells were incubated for 30 min with 5 μM BODIPY-TR-ceramide (Thermo Fisher Scientific, Waltham, MA, USA) complexed with bovine serum albumin (BSA) in phosphate-buffered saline (PBS) prepared following manufacturer’s instructions. After 30 min, cells were rinsed with ice-cold DMEM/F12 and incubated with fresh medium at 37 °C 5% CO_2_ for a further 30 min. Cells were fixed with 4% paraformaldehyde, blocked with 1% (w/v) non-fat milk powder/PBS and co-stained with mouse anti-c myc antibody (clone 9E10, Sigma Aldrich) and nuclear stain (DAPI (4′,6-diamidino-2-phenylindole) (Thermo Fisher Scientific, Waltham, MA)), and AlexaFluor secondary antibodies (Invitrogen). Alternatively, cells were fixed in ice-cold methanol at − 20 °C for 5 min, permeabilised with 0.1% (v/v) Triton X-100/PBS at room temperature for 5 min, blocked with 1% (w/v) non-fat milk powder/PBS and immunostained with rabbit anti giantin (ab37266, Abcam) and mouse anti-β-COP (ab2899, Abcam) or rabbit anti SERPINH1 (HPA029198, Atlas Antibodies) and mouse anti-c myc antibody (clone 9E10, Sigma Aldrich) and nuclear stain (DAPI (4′,6-diamidino-2-phenylindole) (Thermo Fisher Scientific, Waltham, MA)) and AlexaFluor secondary antibodies (Invitrogen). Cells were imaged using confocal microscopy (Leica TCS SP5 Confocal Laser Scanning Microscope with LASX software). Images were processed in Adobe Photoshop and figures assembled in Adobe Illustrator.

#### SDS PAGE and western blotting

Forty-eight hours after transfection, HEK293 cells were treated with 10 μg/ml cycloheximide for 16 h. Cells were then lysed with NP40 lysis buffer and total protein levels were assessed using protein assay (DC Protein Assay, Bio-Rad Laboratories, Hercules, CA, USA). Fifteen microgrammes of total protein per sample was loaded onto a 4–12% Bis-Tris polyacrylamide gel and proteins separated by electrophoresis at 200 V for 1 h. Proteins were transferred to PVDF membrane, blocked with 1% non-fat dry milk powder/PBS. COPB1-cmyc was detected using mouse anti-c-myc antibody (clone 9E10, Sigma Aldrich) and beta actin (control) was detected using anti-beta actin antibody (clone AC15, Sigma Aldrich). HRP-conjugated secondary antibodies were used and immunobands visualised with SuperSignal West Femto reagent (Pierce) on a Chemidoc XRS+ (Bio-Rad, Hercules, USA). Densitometry analysis was performed in ImageLab software (Bio-Rad, Hercules, USA).

## Results

### Clinical information

#### Family 1

**Fig. 2:**
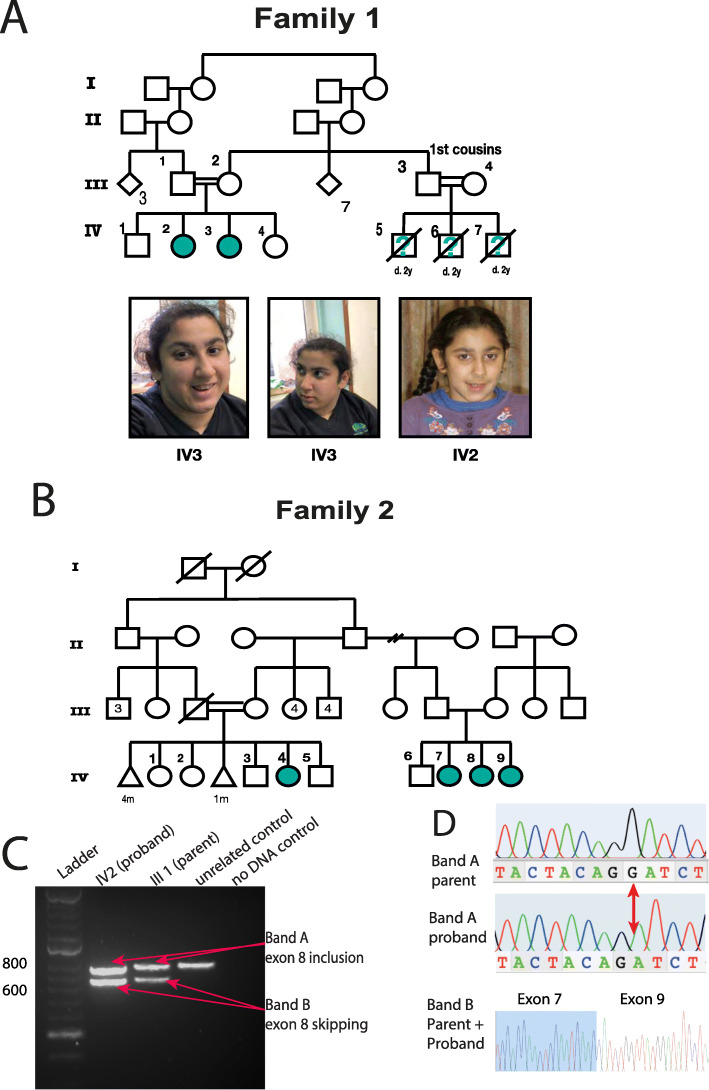
Pedigrees and clinical presentation of individuals with *COPB1* mutations. **a** Family 1 includes two affected individuals IV2 and IV3. Individuals IV5, IV6 and IV7 are also suspected to have been affected with microcephaly and developmental delay. They died in early childhood and no further details were available. IV2 and IV3 have minor dysmorphism with up-slanting palpebral fissures. **b** Family 2 includes four affected individuals from two nuclear families all from the same Saudi tribe. **c** Gel electrophoresis of RT PCR amplicons in Family 1 demonstrates 2 bands in the homozygous state (IV2, an affected individual) and in the heterozygous state (III1, an unaffected parent). **d** Electropherograms of band A and band B from the heterozygous parent and homozygous proband. In the proband’s trace, a G has effectively been deleted due to the creation of a new donor site by the G>T mutation. In band B, Exon 8 has been skipped (proband and parent)

Family 1 are of Roma ethnicity (Fig. 2a). Patient IV2 was the product of an uncomplicated pregnancy and was born at term weighing 3.25 kg (0.2 SD, 50th centile). Antenatal ultrasounds and maternal health were normal. During the neonatal period, she was noted to have torticollis and required physiotherapy. Breastfeeding was normal. She had low tone and came to medical concern at age 8 months as she was not sitting or smiling. Her early milestones were severely delayed (Table 1). She can now go up and down stairs but cannot climb. Her first words were very delayed, occurring at 3 years of age (“Mama”). Her language has subsequently improved and she can speak in short phrases and follow two-step commands. Her speech is unclear but understandable for family members. Her head circumference is − 2.6 standard deviations (SD). Socially she is fully dependant on her parents (Table 1).

**Table 1:** Growth and development

	Family					
	Family 1		Family 2			
Ethnicity	Roma (Polish)		Arab (Saudi)			
*COPB1* variant	NM_016451.4: c957+1G>T (homozygous) GRCh37:g.14504577C>A		NM_016451.4: c.1651T>G p.(Phe551Val) (homozygous) GRCh37:g.14496127A>C			
Patient ID	IV2	IV3	IV7	IV8	IV9	IV4
Sex	F	F	F	F	F	F
Age at physical assessment	14y	16y	15y	15y	11y	7y
OFC	− 2.6 SD	− 3.2 SD	− 2 SD	− 0.2 SD	− 7.9 SD	− 4.4 SD
Weight	+ 2.4 SD	+ 2.8 SD	− 3.1 SD	− 2.8 SD	− 3.9 SD	− 3.2 SD
Height	− 0.1 SD	− 1.9 SD	− 1.8 SD	− 1.2 SD	+ 0.6SD	− 2.2 SD
Neonatal issues	Hypotonia, torticollis	Hypotonia	Unknown	Unknown	Unknown	Unknown
Other genetic findings	Normal microcephaly and cataract panels Balanced 12;13 insertional translocation	Normal microcephaly and cataract panels Unbalanced 12;13 insertional translocation (761 Kb duplication of 13q13.3) Homozygous for *GJB2* c.71G>A	Normal immunodeficiency gene panel	Normal immunodeficiency gene panel	Normal immunodeficiency gene panel	Normal immuno-deficiency gene panel Normal array CGH
Early development	Smiled at 13m Sat at m Walking at 5y First words 3y	Smiled at 13m Sat at 12m Walking at 4y	Early milestones not available Cannot walk	Early milestones not available Cannot walk	Early milestones not available Cannot walk	Early milestones not available Cannot walk
Age at last developmental assessment	18y	16y	17y	15y	11y	11y
Intellectual disability	Severe	Severe	Severe	Severe	Severe	Severe
Language and hearing	Short phrases (unclear language) Follows simple instructions	*GJB2*-associated deafness No speech Difficulty expressing needs	No speech	Language “fair”	No speech	Limited speech
Gross motor	Can go up stairs Cannot climb	Difficulty on stairs Poor balance	Uses wheelchair	Uses wheelchair	Unable to sit or stand	Unable to sit or stand
Social development and behaviour	Unable to perform self-care Poor safety awareness Intermittent incontinence (mostly urinary)	Unable to perform self-care Incontinent of urine and faeces	Fully dependant	Fully dependant	Fully dependant	Fully dependant

Shows subjects’ growth and development. Five of six subjects had microcephaly (− 2 SD); 3/6 had severe microcephaly (> 3SD). Patients in Family 1 developed disproportionate weight to height and a “metabolic syndrome” phenotype (see Table 2). In contrast, in Family 2, subjects were underweight. All participants have a severe intellectual disability. Gross motor abilities are variable with one participant in family 1 able to walk up stairs and two participants in Family 2 unable to sit

**Table 2:** Medical information

	Family					
	Family 1		Family 2			
Ethnicity	Roma (Polish)		Arab (Saudi)			
*COPB1* variant	NM_016451.4:c.957+1G>T (homozygous) GRCh37:g.14504577C>A		NM_016451.4: c.1651T>G p.(Phe551Val) (homozygous) GRCh37:g.14496127A>C			
Patient ID	IV2	IV3	IV7	IV8	IV9	IV4
Eye disease	Unilateral nuclear sclerotic cataract noted at 13y with dense amblyopia Divergent squint	Bilateral cataracts noted age 7y, lens aspiration and intraocular implants at age 8y	Developed cataracts	Developed cataracts	Developed cataracts, intraocular lens in place	Developed cataracts
Endocrine/metabolic disease	Hirsutism, axillary acanthosis, early menarche age 9y Raised BMI	Hirsutism, insulin resistance, axillary acanthosis Raised BMI				
Neurological symptoms		Focal seizures at age 14y, EEG normal, treated with levetiracetam	Spasticity	Spasticity, mostly upper limbs	Spasticity Dystonia	Spasticity
Brain imaging	Normal		Mild generalised atrophy		Normal	
Immune disorders	No	No	Yes	Yes	Yes	Yes
Dysmorphic features	Up-slanting palpebral fissures, high narrow palate, tapering fingers	Up-slanting palpebral fissures, high narrow palate, tapering fingers	Not available	Not available	Not available	Not available
Skin	Large café au lait, striae distensae	Benign nodular prurigo, striae distensae				
Other medical problems	Pes planus Kyphosis and mild scoliosis Migraine	Plano valgus foot deformity Kyphosis				

All subjects from Family 1 and Family 2 developed cataracts. Subjects from Family 1 developed a metabolic syndrome with obesity, hirsutism and insulin resistance or diabetes. Though no major abnormalities were identified on brain imaging, some individuals have neurological symptoms with seizures in one participant (Family 1) and spasticity being present in Family 2. Family 2 also have an immunodeficiency with lymphopenia and inadequate antibody response titres

She has a history of recurrent upper respiratory tract infections and tonsillitis. A sleep study was performed for snoring and pauses in breathing during sleep; this was normal. Initial eye exams were normal. A left-sided nuclear sclerotic cataract was first noted at age 13 years and was associated with the development of a dense amblyopia. A divergent squint was also noted at this time. Menarche was early at 9 years of age. Later in childhood, she developed increased appetite, weight gain, acanthosis nigricans (axillary) and facial hypertrichosis. She has no history of seizures, and CT brain was normal in infancy.

Patient IV3 was also the product of an uncomplicated pregnancy with normal antenatal imaging. She had low tone during the neonatal period. Breast feeding was normal. As with her sister, her early milestones were severely delayed. Her gait is described as unsteady and she has difficulty with stairs. She has planovalgus foot deformity of one foot which is managed with orthopaedic footwear. She was noted to have congenital deafness with 65–90-dB hearing loss associated with a homozygous *GJB2* c.71G>A variant. She has absent speech. She has difficulty communicating her needs especially pain. She understands some simple commands, such as “give it to me”. Initial eye exams were normal. She was noted to have bilateral cataracts at age 7 years which was treated with lens aspiration and intraocular implants at age 8 years. Her vision is normal. Her microcephaly is more severe than her sister’s and currently measures − 3.2 SD.

She developed focal seizures at 14 years. Her interictal EEG was normal, and seizures are controlled with levetiracetam. Menarche occurred at 11 years. As with her sister, she developed increased appetite, increased weight, facial hypertrichosis and axillary acanthosis nigricans later in childhood.

In addition to IV2 and IV3, three first cousins had microcephaly, developmental delay and died early in childhood (IV5, IV6, IV7). No further phenotypic information was available and genetic testing could not be undertaken.

In terms of dysmorphology, both IV2 and IV3 have up-slanting palpebral fissures, narrow palates and tapering fingers.

#### Family 2

Family 2 includes four affected individuals from two nuclear families, all from the same Saudi tribe (Fig. 2b). Prenatal and early neonatal information was not available for this family as subjects were born outside of the hospital. All four individuals have severe intellectual disability. Language impairment is variable in this family (individual IV8 is described as verbally fair at 15 years, IV4 has limited verbal output at 11 years whereas IV9 and IV7 have no language at 17 and 11 years of age respectively). All individuals have severe social impairment and are fully dependant for personal care. IV9 and IV4 have severe microcephaly (> − 3 standard deviations). All four affected individuals have developed cataracts and patient IV9 has had surgical treatment for this. Affected individuals in this family also have poor mobility. They have developed spasticity and have delayed motor skills. IV7 and IV8 use a wheelchair whereas IV9 and IV4 cannot sit or stand. IV9 has dystonia.

Immunodeficiency was also identified in Family 2 with severe lymphopenia being present in all four patients. Specifically, T cell lymphopenia was present with very depressed CD3+CD4+ cells. Immunoglobulin levels were normal overall; however, the patients failed to mount antibody responses to any specific antigens tested. Panel-based genetic testing as part of routine clinical care for known causes of severe combined immunodeficiency did not reveal any putative causal variants of this symptom. More detail on this feature is supplied in Additional file 1: Table S1.

Dysmorphology information/clinical photographs were not available for this family.

### Genetic findings

#### Family 1

Family 1 are known to carry a 12;13 insertional translocation of approximately 761 Kb in size. This is present in a balanced form in individual III2 and individual IV2, as a duplication of chromosome 13q13.3 in individuals IV3 and IV1 (who is phenotypically normal) and as a deletion in 13q3.3 in individual IV4 (who is phenotypically normal). The copy number variant includes *TRPC4* (MIM * 603651) which has not been linked to monogenic disease and *UFM1* (MIM * 610553). Both the deletion and duplication have been classified as likely benign by a clinical laboratory. IV2 was found to have a homozygous *GJB2* c.71G>A variant, established as the cause of her deafness. Gene panel testing for microcephaly (PanelApp Cataracts v2.2, PanelApp Severe Microcephaly v1.47) and cataracts performed on a clinical basis did not reveal a cause for the family’s phenotype.

Whole exome sequencing showed no biallelic predicted pathogenic variants in any known disease genes. (In total, 18 homozygous variants across 17 genes were identified. The candidate variants and their interpretation are supplied in Additional file 1: Table S2.) Further review of variants in non-MIM annotated genes identified a homozygous c.957+1G>T variant in *COPB1*, which affects the + 1 position of the donor site of exon 8 (Chr11(GRCh37):g.14504577C>A). Parents and unaffected siblings were all found to be carriers of this variant, and IV2 and IV3 are homozygous. Four predictive tools were in agreement, all in suggesting donor site loss [25–28]. SpliceAI additionally predicted a new donor site would be created 1 nucleotide before the WT exon 8 donor site, resulting in a 1 nucleotide frameshift (Table 3).

**Table 3:** Variants identified

Variant/family no.	Variant	Predictive tools		Conservation		gnomAD AF
1	c957+1G>T homozygous GRCh37:g.14504577C>A	Human Splicing Finder Donor site loss (− 26.84) Splice Site Prediction by Neural Network Donor site loss (Mutant score = 0) MaxEntScan Donor site loss (− 78.36) SpliceAI Donor site loss New donor site created causing a 1 nucleotide frameshift				Absent
2	c.1651T>G (p.Phe551Val) homozygous GRCh37:g.14496127A>C	Polyphen-2	Probably damaging	Nucleotide conservation	PhyloP 5.21 (strongly conserved)	Absent
		SIFT	Deleterious	Amino acid conservation	High, to Baker’s Yeast ConSurf 8/9	
		CADD (Phred)	32			

The variant identified in Family 1 affects the +1 donor position of exon 8. Note the results from SpliceAI which predicts both donor site loss and creation of a new donor site (verified by experimental data in Fig. 2). The missense variant in Family 2 is predicted to be deleterious by a number of tools

Modelling of the mutation onto the 3D structure of β-COP protein in complex with other components of the COPI coat leaf in complex with the ArfGAP2 uncoating factor (pdb file 5NZS) was performed [11].

**Fig. 3:**
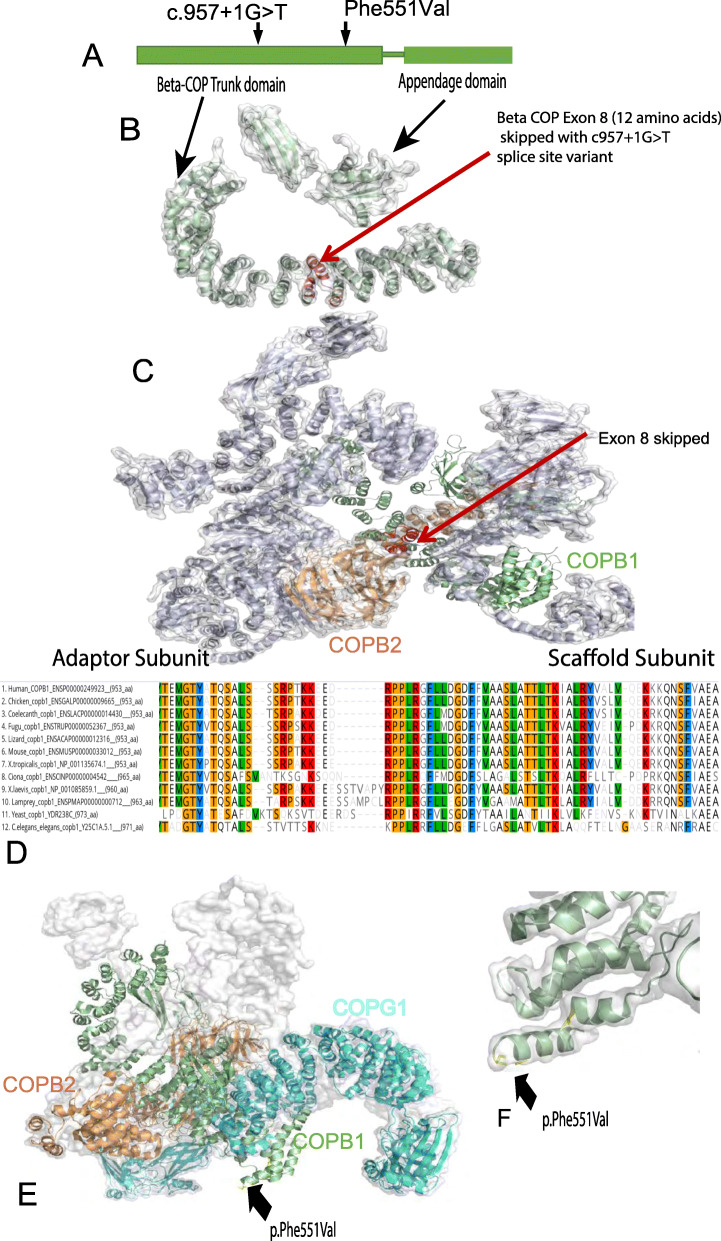
Structure and conservation of *COPB1* protein, and structural effect of missense variants. **a** Simple schematic diagram of COPB1 (β-COP) structure showing two main structural domains; trunk domain and appendage domain, and relative location of the c.957+1G>T and p.Phe551Val variants located towards the N terminal of the trunk domain. **b** COPB1 (β-COP) structure with the location of the 12 amino acids (in red) which are deleted due to exon 8 skipping caused by c957+1G>T. **c** COPB1 (β-COP) structure in context of COPI complex. Note exon 8 (red) makes up an important link between the scaffold and adaptor subcomplexes. **d** Alignment of COPB1 amino acid sequence from *H. sapiens* to yeast showing very high conservation of Phe551 across all species tested. **e** 3D structural modelling of COPB1 in complex with COPB2 and COPG1 showing the location of Phe551 near the site of interaction with COPG1. **f** Higher resolution image showing the location of Phe551Val mutation in a turn in the trunk domain

This splice site mutation lies in the trunk domain β-COP but not at the ARF1 binding site. It leads to exon 8 skipping which causes a 108-bp deletion, and an in-frame deletion of 36 amino acids, resulting in the loss of a helix-turn-helix motif from the alpha solenoid structure (trunk) of β-COP (see Fig. 3b). It is noteworthy that this lost motif in the β-COP trunk lies at a small interface between β-COP and β’-COP (COPB2) (Fig. 3c). This is one of the linking points between the adaptor and scaffold coat subcomplexes composing the COPI complex.

To verify bioinformatic predictions, RT PCR, gel electrophoresis and Sanger sequencing of *COPB1* amplicons were performed on cDNA produced from blood-extracted RNA from a homozygote and heterozygote. Both the homozygous proband (IV2) and the heterozygous parent (III1) cDNA amplified 2 bands (A and B) (Fig. 2c). Sequencing of these bands showed that in both the proband and parent, band B was a PCR amplicon from a transcript with in-frame skipping of exon 8. Band A in the parent was a PCR amplicon of the wild-type transcript with exon 8 inclusion, whereas band A in the proband was a PCR amplicon of a mutant transcript in which exon 8 was included with a 1-bp deletion of c.958G. This causes a frameshift, leading to a new in-frame stop codon (TAA) 18 bp downstream of this deletion (and 18 bp downstream of the junction between exon 7 and exon 8). The mechanism through which this mutant transcript escapes nonsense-mediated decay (NMD) in the homozygote proband may be related to the fact that the premature stop codon is only 18 nucleotides downstream of the exon7/exon8 junction, as previous work has shown that premature stop codons less than 50 nucleotides from an exon/exon boundary escape NMD as they are not recognised by the exon junction complex in the NMD pathway [46]. However, why the transcript undergoes NMD in the heterozygote parent (we did not observe this transcript in the parent) is unclear.

#### Family 2

WGS was undertaken in all affected family members and several unaffected relatives (8 individuals). Only one appropriately segregating homozygous variant was identified, underpinning the power of sequencing multiple family members in large pedigrees [23]. The identified mutation was a homozygous *COPB1* missense variant, c.1651T>G in exon 14 causing a Phe551Val amino acid substitution (Chr11(GRCh37):g.14496127A>C), falling in the β-COP-trunk domain (Fig. 3a). This variant was predicted by PolyPhen 2 to be probably damaging, predicted by SIFT to be deleterious and predicted by CADD (PHRED score) to be deleterious, scoring 32 (Table 3) [39–42].

Phe551 is a highly conserved residue, conserved from human to *C. elegans* and *S. cerevisiae* (Fig. 3b), with a high PhyloP score of 5.21(− 14.1;6.4), modest Grantham score at 50 (0–215) and high ConSurf score of 8/9. Allele frequency was assessed using the gnomAD database. The Family 2 variant (c.1651T>G, p.Phe551Val) was absent.

Modelling of this mutation onto the 3D structure of β-COP protein in complex with other components of the COPI was performed [11]. This showed that the mutation falls within the trunk domain, but lies distant to its binding site with ARF1 and COPI subunits (Fig. 3c). Its significance is more likely to be its effect on internal β-COP structural integrity. Phe551Val lies at a turn or a loop connecting two alpha helices which may influence the directionality of the following helix or the folding of the protein (Fig. 3d). Further functional work was undertaken to investigate these predictions, see the “Modelling of Family 2 missense variant” section.

### copb1 disruptions in *X. tropicalis*

In order to investigate the phenotypic effect of β-COP truncation, we used CRISPR/Cas9 to introduce insertions and deletions (indels) into genomic *copb1* in the western clawed frog *X. tropicalis* at a site adjacent to the genomic location of the variant found in participating Family 1. There is now a large body of evidence showing that making targeted indels in the western clawed frog is so efficient that it is possible to analyse the resulting phenotypes in founder animals [47–53]. This results in robust but more rapid testing of the causality of a gene variant in disease than waiting for F1 or F2 animals to be available. The human and *X. tropicalis copb1* genes share exon structures in the updated frog genome (v10, Xenbase), their COPB1 proteins are > 95% identical (Fig. 3d, Additional File 1: Fig. S1a) and both regions containing the patient variants are conserved.

**Fig. 4:**
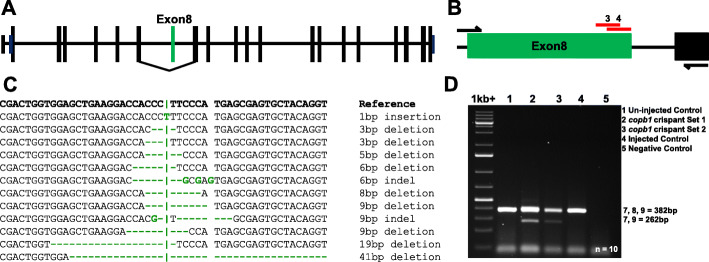
Targeted CRISPR/Cas9 disruption of *copb1* exon 8 generates a range of insertion-deletion changes in vivo. *Xenopus tropicalis copb1* has 21 exons, including an untranslated first exon (**a**). CRISPR/Cas9 directed indel formation disrupting *X. tropicalis* exon 8 using 2 sgRNAs (red: sgRNA3 and sgRNA4 (**b**)) results in homozygous frameshift and splice changes akin to those identified within the patient subpopulation. Genotyping analysis of *X. tropicalis* crispants details the range of indels across three groups of 10 pooled tadpoles (NF41) injected with sgRNA3 following PCR amplification and Sanger sequencing of subclones (**c**). The 41-bp deletion observed in gDNA subclones extends into the intronic region and is proposed to induce exon 8 skipping in crispant *X. tropicalis* tadpoles. Total RNA was obtained from 4 groups (un-injected control (1), copb1 sgRNA 3 crispant set 1 (2) and copb1 sgRNA 3 crispant set 2 (3), injected control (4)) of 10 pooled individuals as outlined in Guille 1999 [51] and cDNA synthesised using Primer Design’s Reverse Transcription Premix 2. Amplification across the *copb1* region of interest revealed a band at 382 bp (exons: 7, 8 and 9) and an additional band in crispants at 262 bp (exons: 7 and 9, Additional file 1: Fig. S1D)

To mimic the Family 1 human genomic change in *Xenopus* as closely as possible (Family 1; exon 8), sgRNAs were designed to create double-stranded DNA breaks (DSBs) at the end of exon 8 in *X. tropicalis copb1* (Fig. 4a, b). Analysis of the target genome region of mosaic, crispant embryos showed both skipping of exon 8 and indels in exon 8 (Fig. 4c, d, Additional file 1: Figure S1) and that both the eyes and the remainder of the tadpole were equally mosaic (Additional file 1: Figure S2). RT-PCR from two separate sets of embryos showed that there was a significant, consistent level of exon skipping across injected embryos (Fig. 4d, Additional file 1: Figure S1). Sanger sequencing of RT-PCR products confirmed the expression of several different transcripts from the targeted region, including transcripts in which exon 8 is skipped, and transcripts in which exon 8 is retained but which contain deletions and insertions (Additional file 1: Figure S1(C,D)). Sequencing of PCR products from genomic DNA from single tadpoles confirmed that the level of mosaicism was high, few cells retained the unaltered DNA sequence at the targeted locus (Additional file 1: Figure S2). PCR products of the CRISPRed locus were subcloned and individually sequenced, showing that a series of deletions had occurred ranging from 5 to 41 bp (Fig. 4c). These large deletions extended well into the intron and were associated with the embryos showing exon 8 skipping.

**Fig. 5:**
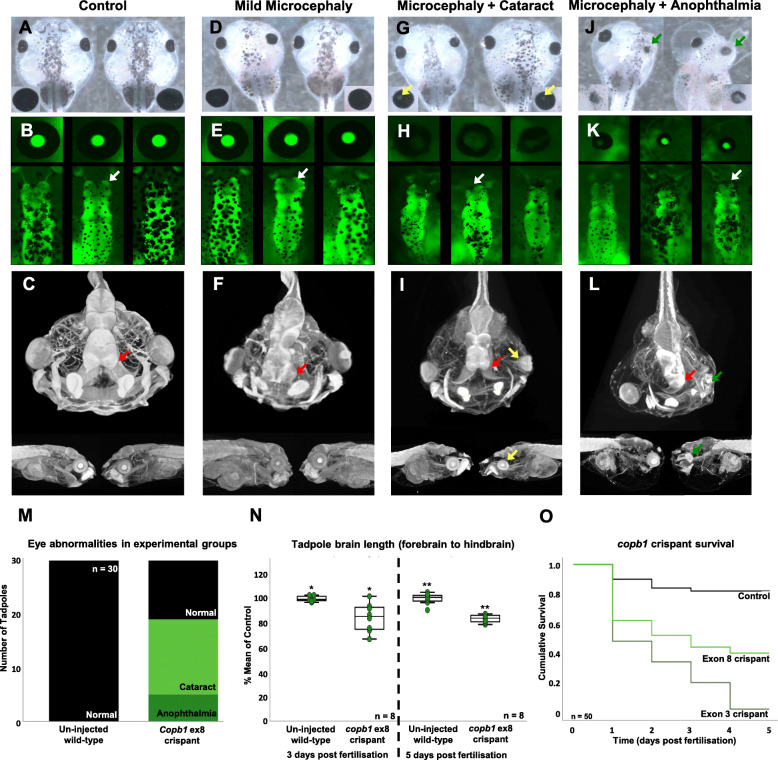
F0 free-feeding wild-type and transgenic [Xtr.Tg (tubb2b:GFP) Amaya, RRID: EXRC_3001] *Xenopus tropicalis* crispants phenocopy key clinical hallmarks. Images show tadpole head structural morphology under normal conditions (**a**–**c**) and across the range of phenotypes observed: mild microcephaly (**d**–**f**), microcephaly with cataract(s) (**g**–**i**) and microcephaly with absent or missing eye structure(s) (**j**–**l**). White arrows on GFP fluorescence images show normal forebrain structures in un-injected control animals (**b**) and altered forebrain structures in mutants, with an increasing severity of microcephaly (**e**, **h**, **k**). Red arrows demonstrate the same forebrain structural trends in higher resolution MicroCT imaging (1% phosphotungstic acid contrast stain: **c**, **f**, **i**, **l**). Cataract formation is indicated in bright-field and MicroCT images by yellow arrows (**g**, **i**) and can be seen as a loss of GFP expression in fluorescence imaging (**h**), whilst green arrows highlight absent or missing structures of the eye (**j**, **l**). Re-occurring eye abnormalities were documented in 30 un-injected and 30 crispant tadpoles (exon 8, sgRNA3) to show the prevalence of cataract formation (mean, 14 tadpoles) and missing eye structures (mean, 5 tadpoles (**m**)). Further, 8 transgenic *X. tropicalis* crispant tadpole brains were imaged and measured (mm) 3 days post-fertilisation (control mean 1 mm, SD 0.02; Crispant mean 0.845 mm, SD 0.12 (*t* = 3.701; *p* = 0.007)) and 5 days post-fertilisation (control mean 6.37 mm, SD 0.29; Crispant mean 5.34 mm, SD 0.20 (*t* = 8.317; *p* = 0.000)) revealing sustained, significant reduction in brain length. Brain length measured as the distance from the forebrain to the hindbrain (Additional file 1: Fig. S5a) was expressed as a percentage of the mean of the control (*N*). Kaplan-Meier survival analysis of 50 un-injected control (median survival time 4.4 days) and crispant *X. tropicalis* tadpoles, injected at the one-cell stage with either sgRNA1 (exon 3 (Additional file 1: Fig. S4, median survival time 2 days) or sgRNA3 (exon 8, median survival time 3 days) show a significant difference in survival by log-rank comparison *p* = 0.000 (**o**)

Two sgRNAs targeted the exon and each produced identical phenotypes in independent experiments, showing that the phenotype was not due to off-target DSBs. Comparing the images from Fig. 5d, g and j with those from Additional file 1: Figure S3 for the crispants makes this clear, with unilateral cataracts, microcephaly and occasional anophthalmia in both sets of tadpoles. The chosen sgRNA(s) were tested for potential off-target sites using CRISPRScan and Cas-OFFinder, neither detected any significant hits. Since these programmes both use previous versions of the genome, we repeated this process manually using BLAST on *X. tropicalis* genome version 10. In this case, a single potential off-target site was detected, but in an intergenic region. Together with the experimental evidence above, this shows that the observed phenotype is due to cutting of the target locus.

We compared the effects of introducing indels into exon 8 with those of introducing indels into exon 3 (Additional file 1: Figure S4(B,C)), where we hypothesised the effect of truncating the protein would be a more complete knockout, whether by nonsense-mediated decay of the *copb1* transcript or by an earlier truncation of the protein. When introduced into exon 3 rather than 8, indels were more damaging to the embryo as evidenced from the survival curve (Fig. 5o) and images of the affected embryos (Additional file 1: Figure S4A). Whilst we cannot formally exclude the possibility that more out of frame indels occur in exon 3 than in exon 8, thus leading to the increased effect observed, the limited sequence data available (Fig. 4c and Additional file 1: Figure S4D) do not support this hypothesis strongly.

All experiments were replicated in embryos from at least three different females and the phenotype data shown are from embryos injected with CRISPR/Cas targeting exon 8 in one cell of a dividing, two-cell embryo. This results in the effect of the protein truncation or internal deletion being focused on one side of the injected embryo along the left-right axis with the other half acting as an internal control [38]. Detailed labelling of structures in tadpoles visualised using MicroCT and in the fluorescent tadpole brain are shown in Additional file 1: Figure S5. The crispants showed a range of deformities, including mild forebrain phenotypes that were only visible in transgenically labelled brains or by MicroCT imaging (Fig. 5): compare the forebrain structures shown by the white arrows in Fig. 5b with Fig. 5e and the red arrows in Fig. 5c with Fig. 5f. More obvious phenotypes include clear microcephaly in 27/30 crispants compared with 2/30 controls (telencephalon to cerebellum length for controls median 0.995 mm ± range of 0.03 mm; crispants median 0.855 mm ± range 0.17 mm; see Fig. 5n). Also, compare the neural structures highlighted by white arrows in Fig. 5b with those in Fig. 5h and Fig. 5k and the red arrows in Fig. 5c with those in Fig. 5i and Fig. 5l. Cataracts or anophthalmia were also observed in 19/30 embryos but were absent in controls (Fig. 5m; highlighted by the yellow arrows in Fig. 5g and i) and revealed by the loss of the fluorescence from the transgenic marker crystallin-GFP in the lens (compare the close-up images of the eyes in Fig. 5b, h). The most extreme phenotypes have a wide range of deformities that include almost complete loss of the eye (see the green arrow in Fig. 5j, l). The few surviving embryos when the gene was disrupted by indels in exon 3 also showed similar, but generally more exaggerated phenotypes (Additional file 1: Figure S4(a,d)). As with the human presentation, the phenotype was specific when taken as a whole, but variable on the individual level.

### Modelling of Family 2 missense variant

We hypothesised that the Family 2 variant (c.1651T>G, p.Phe551Val) affects the local structures and folding of β-COP, which could affect its overall affinity of interactions within COPI complex and undertook in vitro analysis to assess this further.

**Fig. 6:**
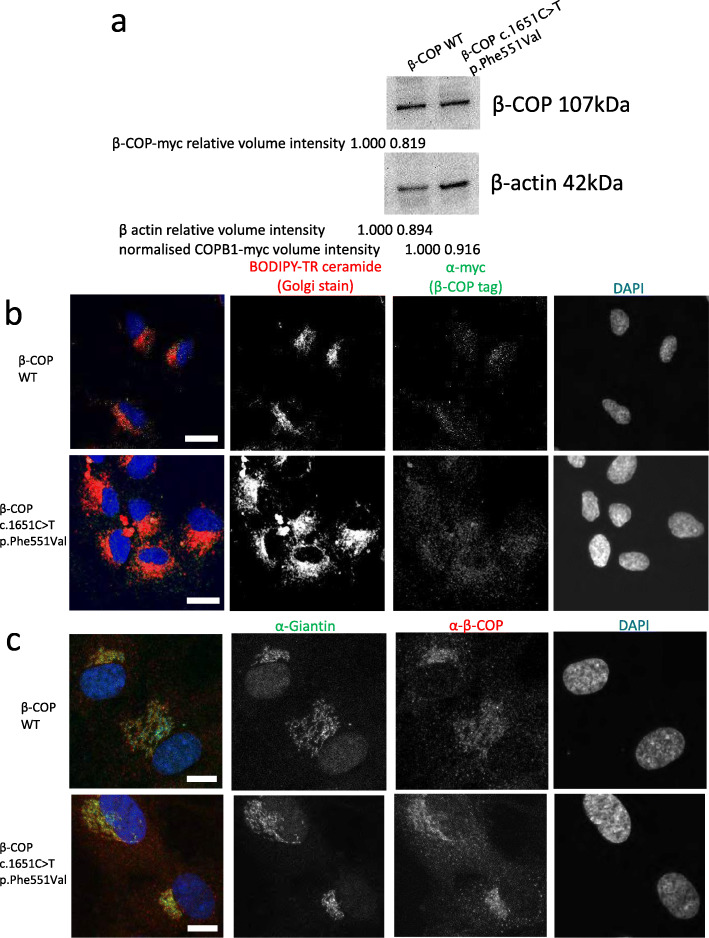
Effect of expression of *COPB1* mutation on in vitro localisation and stability of β-COP protein. **a** Western blot image of protein extracts from HEK293 cells transfected with wild-type, and c.1651T>G (COPB1 tagged with c-myc, immunoblotted with c-myc antibody to detect COPB1, and with beta-actin antibody as loading control). c.1651T>G shows a slightly reduced normalised level. **b** Immunofluorescence images of hTERT-RPE1 cells nucleofected with wild-type *COPB1* (top row), and *COPB1* c.1651T>G p. p.Phe551Val (lower row) tagged with c-myc. Cells were stained with Golgi stain Bodipy-TR-ceramide (red), nuclear stain DAPI (blue) and immunostained with anti-myc (green). Mutant expression shows more diffuse β-COP staining throughout the Golgi and outside the Golgi in the wider cytoplasm. Scale bar = 5 μm. **c** Immunofluorescence images of hTERT-RPE1 cells nucleofected with wild-type *COPB1* (top row), and *COPB1* c.1651T>G p. p.Phe551Val (lower row) tagged with c-myc. Cells were stained with nuclear stain DAPI (blue) and immunostained with an antibody to resident Golgi protein giantin (green) and anti-β-COP (red). This clarified that wild-type β-COP tended to localise to the edges of the Golgi stacks, whereas mutant β-COP was localised throughout the Golgi. Scale bar = 10 μm

Mutant *COPB1* cmyc-DDK-tagged plasmids were generated using site-directed mutagenesis and successful mutagenesis was confirmed using Sanger sequencing. These plasmids were transfected into HEK293 cells, which were then treated with 10 μg/ml cycloheximide to inhibit protein synthesis to provide a more robust test of protein stability. Cells were lysed and protein extracted after 16 h and analysed by SDS PAGE and western blotting. Only a very subtle difference between β-COP-cmyc band intensity was observed between WT and mutant protein (Fig. 6a). Densitometry analysis revealed that relative, normalised levels of β-COP c.1651T>G p.Phe551Val (normalised to beta actin loading control) were lower than β-COP WT levels, suggesting a mild reduction in protein stability of the mutant β-COP (Fig. 6a).

Plasmids were also transfected into hTERT RPE-1 cells and after 48 h the Golgi was stained with BODIPY-TR-ceramide and cells were fixed and β-COP-cmyc was immunostained with anti-cmyc. Confocal microscopy imaging showed a subtle change in localisation of mutant β-COP compared to WT β-COP. Whilst WT β-COP could be seen in a defined pattern at the Golgi, mutant β-COP could be seen in a more diffuse location (Fig. 6b), suggestive of a defect in Golgi to ER recycling of the COPI complex. To investigate this further, we repeated transfections and immunostained the Golgi membrane with an antibody to giantin, which is a protein integral to the Golgi membrane, and co-immunostained with an antibody to β-COP. This showed again that β-COP c.1651T>G p.Phe551Val is more widely distributed throughout the cell, but also more widely distributed throughout the Golgi (Fig. 6c). Whereas wild-type β-COP localised specifically to the edges of the Golgi stacks, mutant β-COP is localised throughout the Golgi, as can be seen by the greater overlap of green (giantin) and red (β-COP) staining, which appears yellow (Fig. 6c). Staining of the ER with anti SERPINH1 showed no difference in localisation of wild-type β-COP and β-COP c.1651T>G p.Phe551Val relative to the ER (Additional file 1: Fig. S6). Together, these data suggest that β-COP c.1651T>G p.Phe551Val is defective in Golgi to ER recycling, with this mutant β-COP getting retarded in the Golgi.

## Discussion

In this paper, we present a new syndrome characterised by severe developmental delay and cataracts with the majority of patients also being microcephalic. Family 2 also exhibits spasticity and immunodeficiency, whereas Family 1 demonstrates a “metabolic syndrome” phenotype from late childhood. Our model reflects this highly heterogenous phenotype with some *Xenopus* showing neither microcephaly nor cataracts, with others being severely affected. Our in silico and in vitro missense modelling work demonstrates a high conservation of the specific residue (c.1651T>G p.Phe551Val) mutated in this family and an impact on localisation of protein with this specific mutant residue observed in cells, with this mutant protein being retarded in the Golgi. Given that the primary role of COPI is to return ER-resident molecules from the Golgi, the localisation abnormality seen associated with the expression of the missense construct is likely to have a significant effect on normal functioning of the endomembrane system. As the splice variant in Family 1 causes skipping of a 108-bp exon, leading to an in-frame deletion of 36 amino acids in the protein, we hypothesise that both the missense and splice variants are hypomorphs which retain some functionality. We further hypothesise that null alleles may be embryonically lethal in mammals, as for other some coatomer subunits [3, 4]. This hypothesis is supported by data from *Xenopus* in which frameshift indels in exon 3 of *copb1* were introduced mosaically using CRISPR/Cas. Survival rates were very low in these knockout animals and survivors’ phenotypes similar to, but generally more severe than those in embryos carrying indels in exon 8.

Microcephaly was a prominent phenotype in this syndrome and clearly reproduced in the *Xenopus* model. A homozygous variant in the related *COPB2* (β’-COP) has been linked to a primary microcephaly syndrome [4]. The authors describe siblings with microcephaly, failure to thrive, spasticity, cortical blindness and severe developmental delay, which share some obvious similarities to, in particular, Family 2 in our study. *ARCN1-*related syndrome (δ-COP) also includes features of microcephaly and, albeit milder, developmental delay. Interfering RNA screens indicate that *COPB1* may play an important role in cell division which could be linked to the microcephaly seen in these patients [54]. Previous studies have indicated that COPI has numerous roles in neuronal function. For example, it may play a role in the transport of RNA in neurons and is also involved in the transport of SNARE complex proteins which traffic neurotransmitters [55, 56]. Interferonopathies are a known cause of microcephaly, and notably a recent study investigating the protein network of interferon-stimulated genes identified β-COP as one of the two most connected proteins in this network of 1400 [57].

All affected individuals in this study had markedly delayed gross motor milestones. Interestingly, COPI (including β-COP) interacts with the protein Staismon which is thought to be important in synaptic transmission in motor circuit neurons and in motor axon outgrowth [58]. Additionally, COPI, through its subunit α-COP, binds with survival motor neuron protein (SMN), which is deficient in spinal muscular atrophy (SMA) and is involved in its axonal transport [59]. Furthermore, SMA patients show abnormal Golgi apparatus morphology which can be corrected by overexpression of α-COP [60].

Six of six individuals identified in this study have developed cataracts. Interestingly, the cataracts in Family 1 developed quite late in childhood, which is a less common presentation for congenital cataracts. Several other coatopathies demonstrate cataracts in their phenotype. For example, cranio-lenticulo-sutural dysplasia has been linked to homozygous variants in the COPII gene *SEC23A*. These patients had a missense mutation in SEC23A (use italics for this word please) which, when exogenously expressed in cells in culture, displayed a cellular phenotype suggestive of abnormal Golgi to ER trafficking [61]. The pathogenesis of this phenotype is unclear.

Family 2 exhibits the additional phenotype of immunodeficiency; this was absent from Family 1 who have had normal immunology studies. Given the role of coat proteins in the secretory pathway, we hypothesise altered immune function may be, at least in part, due to the high demand of plasma cells on protein (immunoglobulin) expression, modification and secretion. Additionally, β-COP is listed as a secretory granule membrane protein with a role in neutrophil degranulation on reactome database [62]. We also note the role of β-COP in MHC1 and CD4 degradation in HIV-infected cells [63, 64]. It is also possible that the additional feature of immunodeficiency and spasticity may be due to an as yet unidentified second variant in this highly selected family though no appropriate candidates were identified on extensive review.

## Conclusions

In conclusion, we have described a novel recessive syndrome caused by biallelic variants in *COPB1.* Disrupting *copb1* in *Xenopus* clearly shows phenotypic changes in common with the patients, in particular microcephaly and cataracts, strongly supporting the link between the patient variants and their pathology. The utility of these data in making the connection between the gene change, including exon skipping, and phenotype is another demonstration of how successful *X. tropicalis* is as a tool for the characterisation of novel disease genes. Taken together, our data add to the growing body of evidence that COPI subunits are essential for brain development and human health.

## Supplementary Information

**Additional file 1: Fig. S1**. Demonstrates how CRISPR/ cas9 genome editing induces exon skipping in *X.tropicalis*. **Fig. S2.** Shows the eye (a target organ) and the remainder of the crispant tadpole are equally mosaic. **Fig. S3.** Confirms that disruption to *copb1* exon 8 in *X.tropicalis* mirrors syndromic hallmarks. **Fig. S4.** Supplemental figure demonstrates a more exaggerated phenotype in tadpoles injected with CRISPR/cas9 targeting exon 3, *copb1.* **Fig. S5.** Illustrates *Xenopus* anatomy and the significant reduction in brain size seen in transgenic tadpoles. **Fig. S6.** Shows there is no difference in localisation of wild type beta COP versus the Family 2 variant beta COP. **Table S1.** Details the immunodeficiency investigations performed in Family 2. **Table S2.** Details other shared homozygous variants identified in both probands of Family one.

## Data Availability

Family 1’s whole exome sequencing data is not publicly available as it was generated in the UK National Health Service as part of the patients’ clinical investigations and cannot be shared outside of this service. Family 2’s whole genome sequencing data cannot be shared as this is not compliant with our IRB approval. The variants identified in both families have been deposited on ClinVar, https://www.ncbi.nlm.nih.gov/clinvar/variation/996016/ [65] and https://www.ncbi.nlm.nih.gov/clinvar/variation/996037/ [66].

## Abbreviations

BSA: Bovine serum albumin
COPI: Coat protein complex 1
COPII: Coatomer protein II
DMEM: Dulbecco’s modified Eagle’s medium
ER: Endoplasmic reticulum
ERGIC: Endoplasmic reticulum-Golgi intermediate compartment
HEK293: Human embryonic kidney 293 cells
NMD: Nonsense-mediated decay
MicroCT: Microcomputed tomography
PBS: Phosphate-buffered saline
PTA: Phosphotungstic acid
RT-PCR: Reverse transcriptase PCR
SD: Standard deviation
SMA: Spinal muscular atrophy
SMN: Survival motor neuron protein
TIDE: Tracking of Indels by Decomposition
WT: Wild type
