# Supplementary material for: Biallelic variants in COPB1 cause a novel, severe intellectual disability syndrome with cataracts and variable microcephaly
Source: Genome Med. 2021 Feb 25;13:34. doi: 10.1186/s13073-021-00850-w (PMC7908744; doi:10.1186/s13073-021-00850-w)
Supplement: Supplementary file 1 — Additional file 1: Fig. S1. Demonstrates how CRISPR/ cas9 genome editing induces exon skipping in X.tropicalis. Fig. S2. Shows the eye (a target organ) and the remainder of the crispant tadpole are equally mosaic. Fig. S3. Confirms that disruption to copb1 exon 8 in X.tropicalis mirrors syndromic hallmarks. Fig. S4. Supplemental figure demonstrates a more exaggerated phenotype in tadpoles injected with CRISPR/cas9 targeting exon 3, copb1. Fig. S5. Illustrates Xenopus anatomy and the significant reduction in brain size seen in transgenic tadpoles. Fig. S6. Shows there is no difference in localisation of wild type beta COP versus the Family 2 variant beta COP. Table S1. Details the immunodeficiency investigations performed in Family 2. Table S2. Details other shared homozygous variants identified in both probands of Family one. [file 13073_2021_850_MOESM1_ESM.pdf]

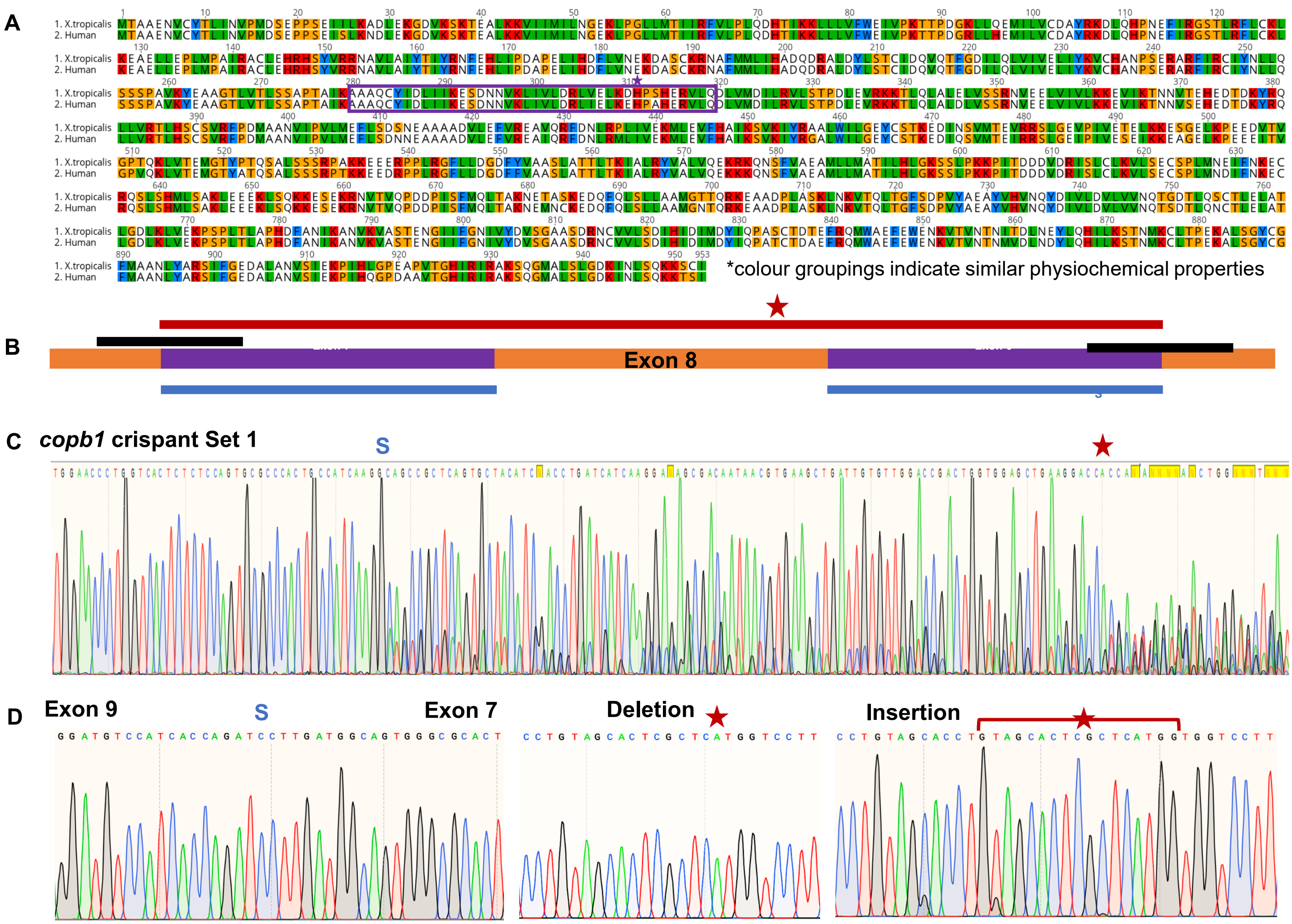

Fig S1 Single guide RNAs induce exon skipping in crispant *Xenopus tropicalis* tadpoles. The *copb1* amino-acid sequence of *H. sapiens* and *X. tropicalis* aligned using MUSCLE (Geneious Prime 2020) shows significant sequence conservation (95.4%) particularly in the region of interest, here the skipped exon 8 is shown within the purple box and the point of indel formation depicted by the star (A). A partial schematic of *copb1*, demonstrates the position of RT-PCR primers spanning exons 6 to 10 (black) and the two alternative outcomes (red/ blue) identified by Sanger sequencing (B). Initial Sanger sequencing traces of amplicons (C) suggested the presence of both indels (red star) and exon 8 skipping (blue 'S') in mosaic crispant tadpoles, these outcomes were confirmed by Sanger sequencing of sub-clones (D).

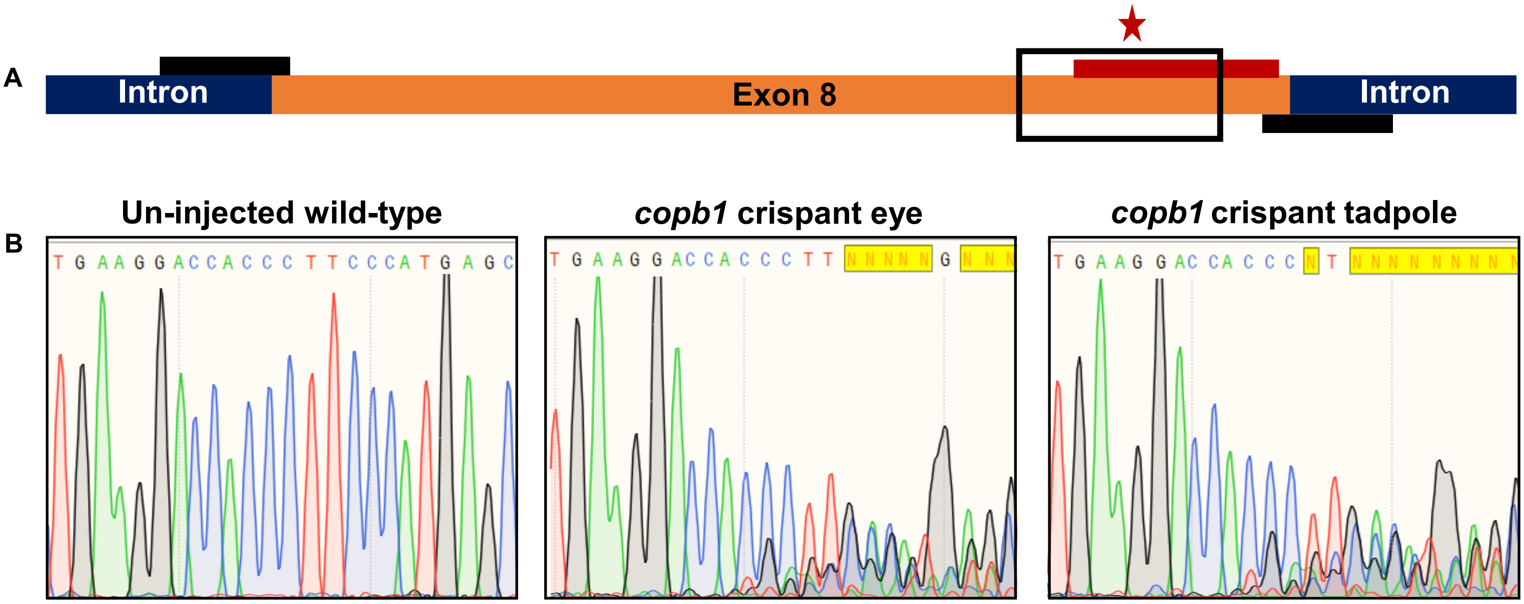

Fig S2 CRISPR/Cas induced indels within exon 8 of *copb1* are similar in the target organ of interest (eye) when compared to genomic DNA extracted from the remaining tadpole. Fertilised *X. tropicalis* eggs from a single female were injected with cas9 protein and sgRNA3 targeting exon 8 of *copb1*, at free feeding stage tadpoles were terminally anaesthetised and the eyes dissected. DNA prepared from the eye and the remaining tadpole was amplified; the amplicon from the eye or tadpole of the crispants was sequenced from the region shown in (A) and demonstrated similar mosaicism in both the dissected eyes and remainder of the tadpole (compare sequence traces in (B)).

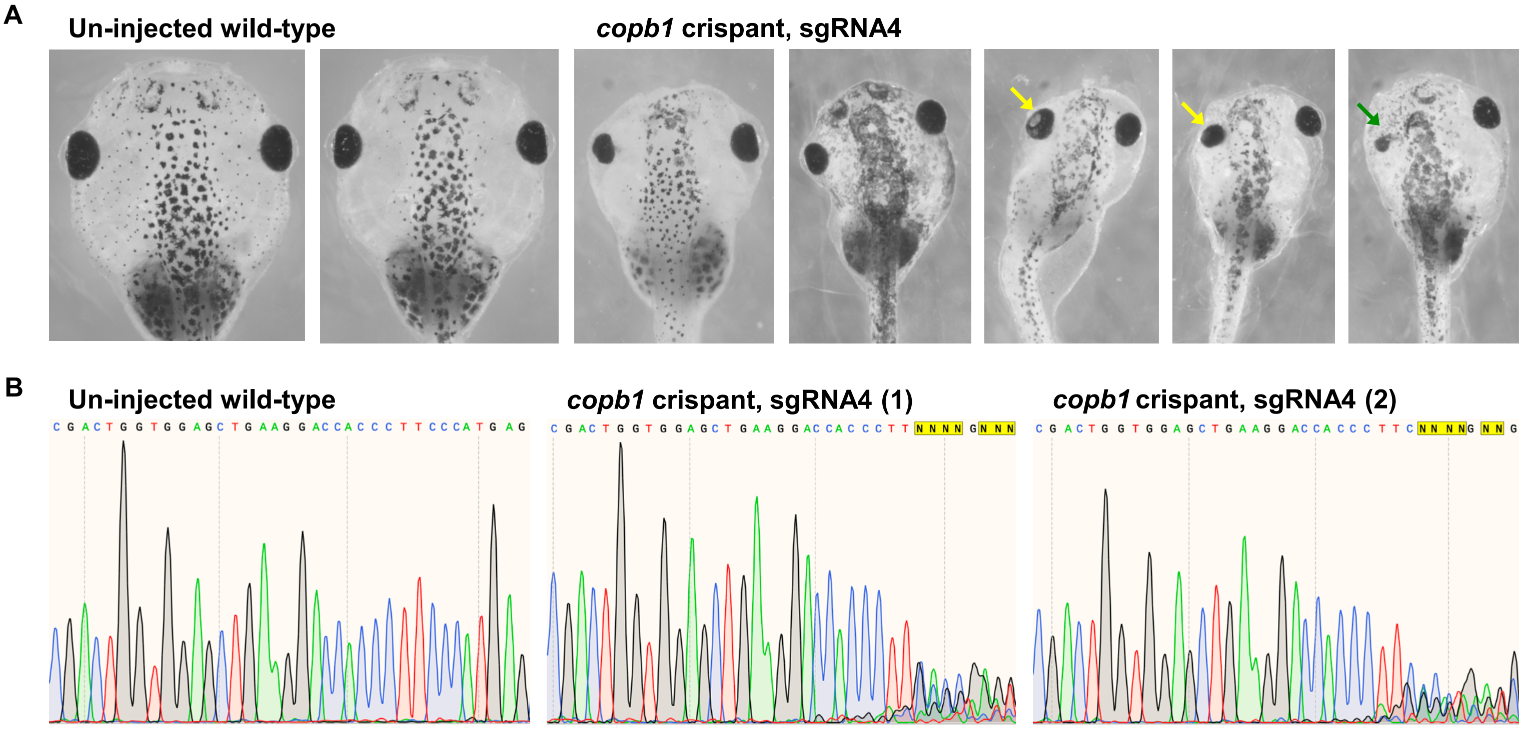

Fig S3 **Disruption to *copb1* exon 8 by sgRNA4 shows the same phenotype as caused by sgRNA3 and reliably replicates the key syndromic hallmarks identified in a patient subpopulation bearing homozygous changes to COPB1.** *Xenopus tropicalis* embryos injected as 1-cell at 2-cell stage with a second sgRNA (sgRNA4) targeting exon 8 show cataract formation (yellow arrow), anophthalmia (green arrow) and microcephaly (A). Confirmation of disruption can be seen in Sanger sequencing traces following PCR amplification of the target region of interest in two crispant tadpoles (B).

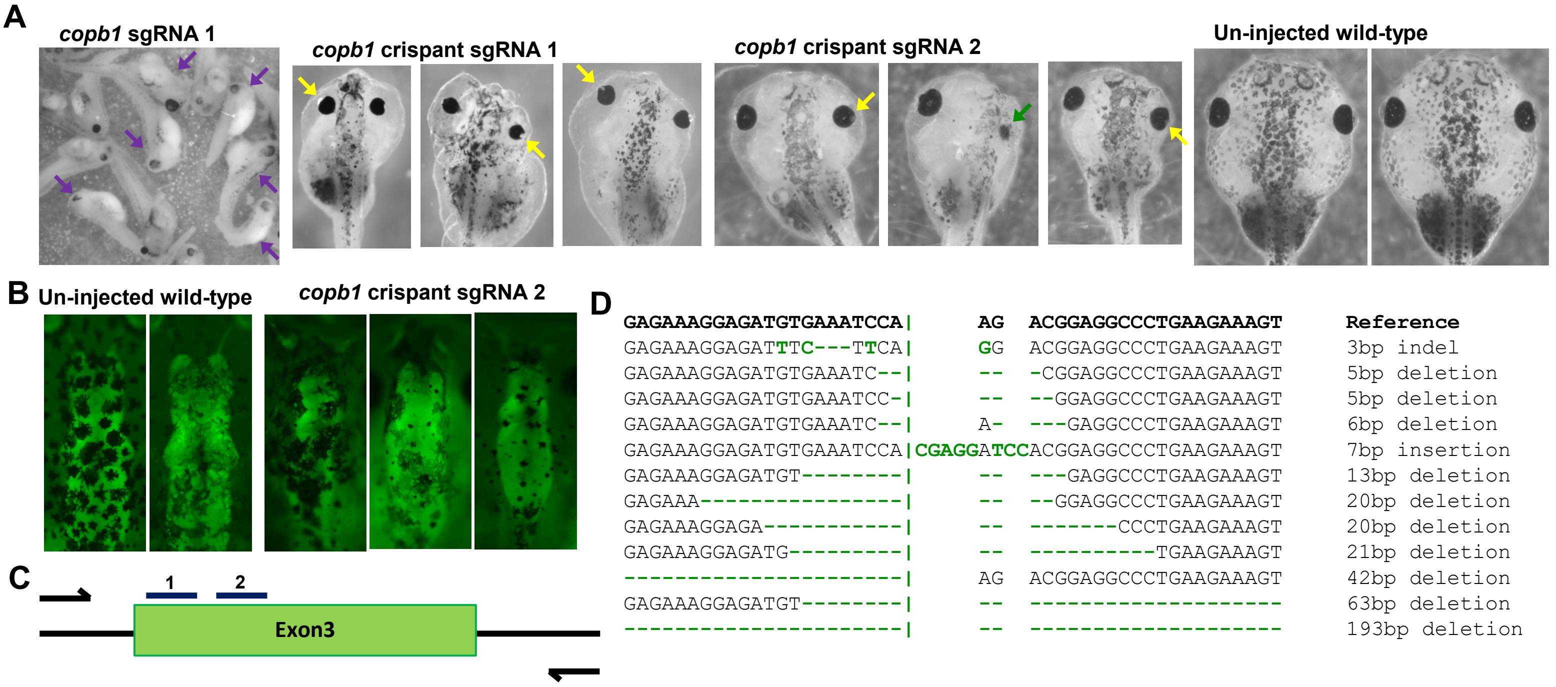

Fig S4 **Disruption to exon 3 of *copb1* in *Xenopus tropicalis* tadpoles, whilst largely lethal, reveals similar syndromic phenotypic traits in highly mosaic F0 survivors.** Disruption to Exon 3, *copb1*, using 2 sgRNAs (sgRNA1 (taatacgactcactataGGGAGATGTGAAATCCAAGAgttttagagctagaa) and sgRNA2 (taatacgactcactataGGGATCATCATGATCCTAAAgttttagagctagaa)) in one of two cells (at two-cell stage) promoted embryo survival to swimming tadpoles stages, circumventing the observed mortality rate in one-cell injected embryos (purple arrow (A)). Surviving individuals were observed to have cataracts (yellow arrows (A)) and anophthalmia (green arrow (A)), in addition microcephaly was evident in bright field images (A) and supported by imaging in a transgenic [Xtr.Tg(tubb2b:GFP)Amaya, RRID: EXRC_3001] background (B). The partial gene schematic depicts placement of sgRNA1, sgRNA2 and the primers (FWD:AGTCAAGCGTCTCCATGGTT; REV:TCACCCACCACCGCTAAAG) used to confirm sequence disruption in gDNA samples (C). Amplicons from 10 pooled individuals collected at NF10 were subcloned to assess the range of in- and out-of-frame indels in crispant tadpoles (D).

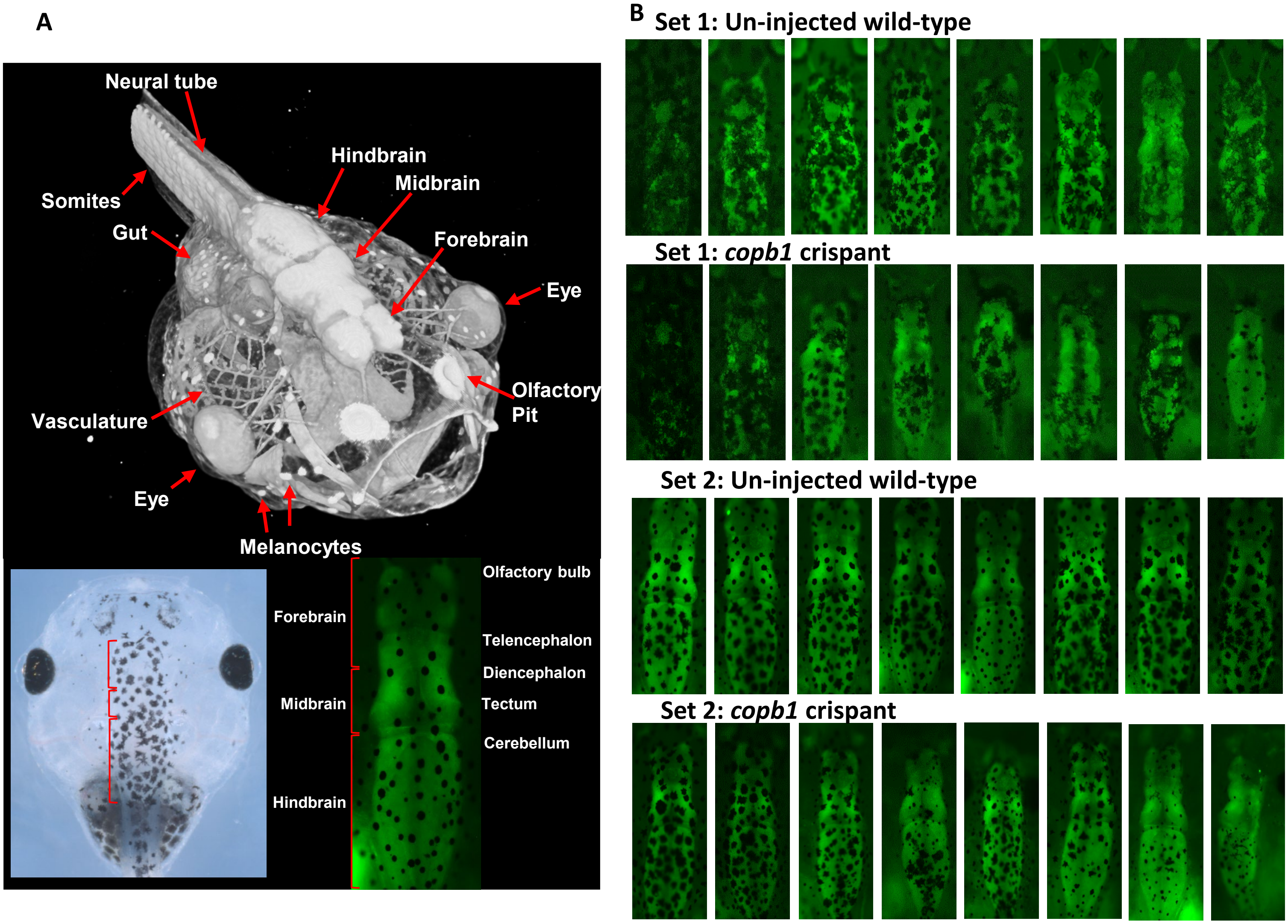

Fig S5 Identification of key anatomical structures in *X. tropicalis* tadpoles. Gross morphologic annotation highlights structures of interest in MicroCT, Bright-field and Transgenic [Xtr.Tg(tubb2b:GFP)Amaya, RRID: EXRC_3001] imaging of uninjected control *X. tropicalis* tadpoles (A). Transgenic *X. tropicalis* crispant tadpoles, with disruption to exon 8 (sgRNA3) within *copb1* show poor development of forebrain regions and sustained reduction in brain length (measured as forebrain to hindbrain (red brackets, A)) at both 3 days post fertilisation (compare Set 1) and 5 days post fertilisation (compare Set 2) (B).

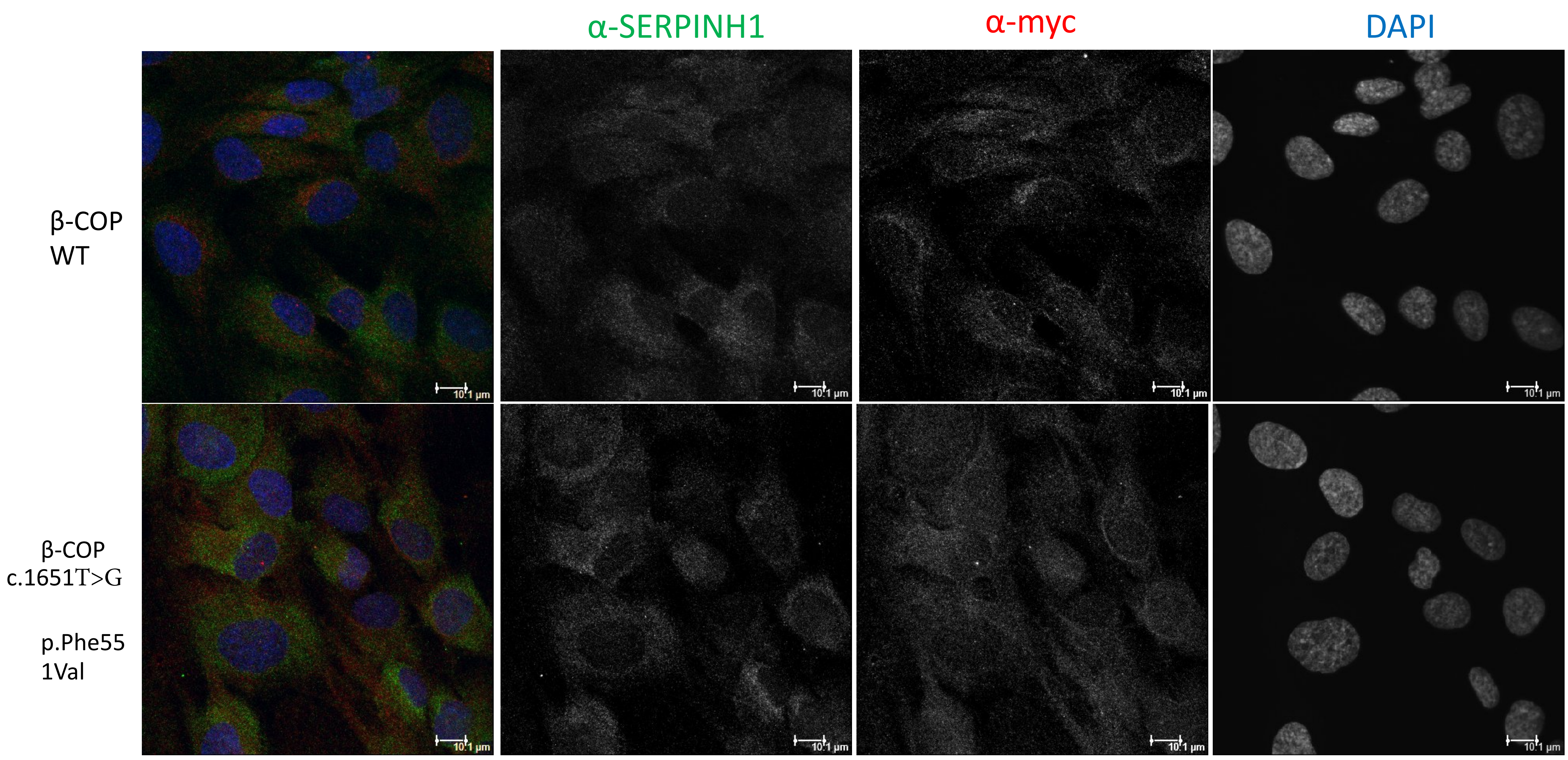

Fig S6 Endoplasmic reticulum immunofluorescence. Staining of the ER with anti SERPINH1 showed no difference in localisation of wild-type β-COP and β-COP c.1651T>G p.Phe551Val relative to the ER.

Table S1

Subjects in Family 2 have severe immunodeficiency. Lymphopenia is noted specifically affecting T cell levels. Patients have normal serum immunoglobulin levels however specific antibody immune responses to all specific antigens are negative post vaccination.

Patient	IV4	IV7	IV8	IV9
WBC Count	1.70 x10^9/L	2.52 x10^9/L	1.63 x10^9/L	1.00 x10^9/L
Lymphocyte Count (cells/mcL)	93	191	489	333
Subsets (cells/mcL)	CD2<br>55.00 (59 %)<br><br>CD3<br>26.00 (28 %)<br><br>CD3+CD4+<br>6.00 (6 %)<br><br>CD3+CD8+<br>19.00 (20 %)<br><br>CD3+CD45RA+<br>22.00 (24 %)<br><br>CD3+CD45RO+<br>72.0 (77 %)<br><br>CD25 5.0 (5 %)<br><br>CD19 29.0 (31 %)<br><br>CD3-CD16+56+<br>43.0 (46 %)	CD2<br>84.00 (44 %)<br><br>CD3<br>66.00 (34 %)<br><br>CD3+CD4+<br>33.00 (17 %)<br><br>CD3+CD8+<br>30.00 (16 %)<br><br>CD3+CD45RA+<br>54.00 (28 %)<br><br>CD3+CD45RO+<br>137.0 (72 %)<br><br>CD25 5.0 (2 %)<br><br>CD19 102.0 (53 %)<br><br>CD3-CD16+56+<br>32.0 (17 %)	CD2<br>425.00 (87 %)<br><br>CD3<br>115.00 (24 %)<br><br>CD3+CD4+<br>57.00 (12 %)<br><br>CD3+CD8+<br>42.00 (8 %)<br><br>CD3+CD45RA+<br>78.00 (16 %)<br><br>CD3+CD45RO+<br>409.0 (84 %)<br><br>CD25 15.0 (3 %)<br><br>CD19 59.0 (12 %)<br><br>CD3-CD16+56+<br>328.0 (67 %)	CD2<br>197.00 (59 %)<br><br>CD3<br>161.00 (48 %)<br><br>CD3+CD4+<br>53.00 (16 %)<br><br>CD3+CD8+<br>72.00 (22 %)<br><br>CD3+CD45RA+<br>109.00 (33 %)<br><br>CD3+CD45RO+<br>223.0 (67 %)<br><br>CD25 8.0 (2 %)<br><br>CD19 107.0 (32 %)<br><br>CD3-CD16+56+<br>53.0 (16 %)
IgG G/L	5.49	4.81	4.97	3.73
IgM G/L	0.76	0.83	0.43	0.55
IgA G/L	0.96	1.88	0.90	0.89
Tetanus Toxoid IgG Antibody IU/ML	0.01	0.00	0.00	0.04

Table S2. Additional shared homozygous variants identified and interpretation.

Gene	Variant	Interpretation
TCN2	Chr22(GRCh37):g.31008867T>C LRG_116:g.10707T>C	Irrelevant phenotype, 40 homozygotes on gnomad
ABCC8	Chr11(GRCh37):g.17436865G>A	Irrelevant phenotype, 160 homozygotes on gnomad
ABCC8	Chr11(GRCh37):g.17491746T>C	Irrelevant phenotype
ACKR2	Chr3(GRCh37):g.42907112A>C	GnomAD exomes allele frequency = 0.434
PROCR	Chr20(GRCh37):g.33764632C>G	GnomAD exomes allele frequency = 0.554
PITRM1	Chr10(GRCh37):g.3208583_3208584insGAGGAATGGCACGCTAGGGAAGGC	Non-coding insertion in gene with irrelevant phenotype
FADS6	Chr17(GRCh37):g.72889676_72889677ins54	In frame insertion in non-morbid gene
MROH8	NG_033795.1:g.5202_5230dup	Non-morbid gene with poorly understood function, very tolerant to LoF (PLI=0)
ASCC2	Chr22(GRCh37):g.30189397T>C	Non-morbid gene involved in DNA damage, conflicting computational predictions
MN1	Chr22(GRCh37):g.28193641G>T	Computational tools predict tolerated, poorly conserved, irrelevant phenotype, inheritance pattern does not fit
DGCR6L	Chr22(GRCh37):g.20306800_20503200dup	Non-coding CNV (duplication) in non-morbid gene
FAM230A	Chr22(GRCh37):g.20306800_20503200dup	Non-coding CNV (duplication) in non-morbid gene
GGTLC3	Chr22(GRCh37):g.20306800_20503200dup	Non-coding CNV (duplication) in non-morbid gene
RIMBP3	Chr22(GRCh37):g.20306800_20503200dup	Non-coding CNV (duplication) in non-morbid gene
TMEM191B	Chr22(GRCh37):g.20306800_20503200dup	Non-coding CNV (duplication) in non-morbid gene
ZNF788	Chr19(GRCh37):g.12222642C>T	Non-morbid gene, variant affects a poorly conserved residue
GCDH	Chr19(GRCh37):g.13002156G>A	Irrelevant phenotype
ZNF490	Chr19(GRCh37):g.12693748C>G	Intronic variant in non-morbid gene
